# Transcriptomic Analysis of Long Non-coding RNA-MicroRNA-mRNA Interactions in the Nucleus Accumbens Related to Morphine Addiction in Mice

**DOI:** 10.3389/fpsyt.2022.915398

**Published:** 2022-06-02

**Authors:** Xiaojie Li, Bing Xie, Yun Lu, Hongyu Yang, Jian Wang, Feng Yu, Ludi Zhang, Bin Cong, Di Wen, Chunling Ma

**Affiliations:** Hebei Key Laboratory of Forensic Medicine, Research Unit of Digestive Tract Microecosystem Pharmacology and Toxicology, Collaborative Innovation Center of Forensic Medical Molecular Identification, College of Forensic Medicine, Chinese Academy of Medical Sciences, Hebei Medical University, Shijiazhuang, China

**Keywords:** morphine, addiction, nucleus accumbens, lncRNA, ceRNA network

## Abstract

Recent research suggest that some non-coding RNAs (ncRNAs) are important regulators of chromatin dynamics and gene expression in nervous system development and neurological diseases. Nevertheless, the molecular mechanisms of long non-coding RNAs (lncRNAs), acting as competing endogenous RNAs (ceRNAs), underlying morphine addiction are still unknown. In this research, RNA sequencing (RNA-seq) was used to examine the expression profiles of lncRNAs, miRNAs and mRNAs on the nucleus accumbens (NAc) tissues of mice trained with morphine or saline conditioned place preference (CPP), with differential expression of 31 lncRNAs, 393 miRNAs, and 371 mRNAs found. A ceRNA network was established for reciprocal interactions for 9 differentially expressed lncRNAs (DElncRNAs), 10 differentially expressed miRNAs (DEmiRNAs) and 12 differentially expressed mRNAs (DEmRNAs) based on predicted miRNAs shared by lncRNAs and mRNAs. KEGG pathway enrichment analyses were conducted to explore the potential functions of DEmRNAs interacting with lncRNAs in the ceRNA network. These DEmRNAs were enriched in synaptic plasticity-related pathways, including pyrimidine metabolism, ECM-receptor interaction, and focal adhesion. The correlation between the relative expression of lncRNAs, miRNAs and mRNAs was analyzed to further validate predicted ceRNA networks, and the Lnc15qD3-miR-139-3p-Lrp2 ceRNA regulatory interaction was determined. These results suggest that the comprehensive network represents a new insight into the lncRNA-mediated ceRNA regulatory mechanisms underlying morphine addiction and provide new potential diagnostic and prognostic biomarkers for morphine addiction.

## Introduction

Opioids, including morphine, are a class of powerful analgesics used to treat various forms of acute and chronic pain. Nevertheless, long-term morphine treatment leads to drug addiction–a chronic, relapsing disease with a complicated mechanism (1). Accumulating research suggests that the development of morphine addiction involves changes in synapse structure and neural plasticity (2, 3). The persistence of these neuroadaptations is regulated in part by epigenetic modification of gene expression programs in the nucleus accumbens (NAc) and other drug reward-associated brain regions (4, 5). Massart et al. determined that cue-induced cocaine seeking is related to modulation of DNA methylation in the NAc (6). Zhang et al. showed that the histone demethylase KDM6B enhances cocaine-induced synaptic plasticity in the medial prefrontal cortex (7).

Among various epigenetic processes, attention has recently been focused on non-coding RNAs (ncRNAs), including microRNAs (miRNAs) and long non-coding RNAs (lncRNAs), which play a important role in various biological functions. Forget et al. reported that the overexpression of miR1 in dorsal striatum D1-striato-pallidal projection neurons reduced cocaine-induced CPP reinstatement (8). LncRNAs are a class of non-coding transcripts over 200 nt long (9). Recently, some studies have demonstrated that it is an essential player in neurodevelopmental (10) and neuropsychiatric disorders, such as addiction. Michelhaugh et al. implicated changes in lncRNA expression levels in heroin addicts (11). In addition, Xu et al. reported that lncRNA Gas5 in the NAc attenuated the response of mice to cocaine administration (12). Nevertheless, research regarding lncRNAs and their potential mechanism in morphine addiction is still in its infancy.

Many functions of lncRNAs have been illustrated, such as interacting with mRNAs (13), binding to transcription factors (14) and acting as competitive endogenous RNAs (ceRNAs) (15). According to the ceRNA hypothesis, lncRNAs regulate target gene expression by competitively binding to miRNA response elements (MREs) (16). Recent evidence has revealed the critical roles of lncRNA-related ceRNA networks in neurological damage and brain disorders. For instance, the interference of BACE1-AS-regulated ceRNA networks could alleviate neuronal damage (17); LncGAS5 promotes the progression of Parkinson’s disease (PD) *via* regulating the miR-223-3p/NLRP3 axis (18). Thus, a comprehensive analysis of the regulatory network of morphine addiction may assist in the identification of potential useful molecules for the diagnosis and therapy of morphine addiction.

In the present research, RNA sequencing technology (RNA-Seq) was used to characterize lncRNA, miRNA and mRNA transcriptome profiles in the NAc tissues of mice with morphine or saline conditioned place preference (CPP) training. The co-regulatory functional ceRNA network was created according to predicting RNA interaction pairs. Gene Ontology (GO) and Kyoto Encyclopedia of Genes and Genomes (KEGG) analyses were conducted to elucidate the biological functions of the differentially expressed mRNAs (DEmRNAs) in the ceRNA network. Additionally, quantitative reverse transcription polymerase chain reaction (qRT-PCR) and correlation analysis with lncRNA, miRNA, and mRNA changes were performed to validate the predicted ceRNA pattern. This research provides a novel insight into the transcriptional processes of morphine addiction and therapeutic targets for the treatment of morphine addiction.

## Materials and Methods

### Animals

Adult male C57BL/6N mice (7–8 weeks old) were purchased from the Beijing Vital River Laboratory. Standard lab chow and water were available *ad libitum*. All animals were housed in a temperature (21 ± 2°C)- and humidity (60 ± 5%)-controlled environment and maintained in a 12 h light/12 h dark cycle (lights on at 19:00). All animal treatments were allowed by the Animal Care and Use Committee of Hebei Medical University. All experiments followed the ARRIVE guidelines.

### Morphine-Induced Conditioned Place Preference

The CPP apparatus consisted of two compartments (15 cm × 15 cm × 20 cm each) with distinct tactile (smooth and rough floors) and visual cues (white vertical stripes and white horizontal stripe walls), which were separated by a board. The unbiased CPP paradigm utilized in this research was modified from previous studies (19, 20). The CPP protocol consisted of four stages: habituation, pretest, conditioning, and test. The animals were individually habituated to the researcher by handling for 3 min, which was repeated for 3 days before the CPP pretest. In the pretest (Day 0), the mice were allowed to move freely and explore the entire equipment for 15 min. The sessions were videotaped, and the time spent in each compartment was determined. Mice demonstrating ≥15% preference for one side in the pretest were excluded from the study (7). During the conditioning phase (Days 1–6), we used two alternative daily conditioning sessions, which lasted 45 min each. The mice were injected with morphine (10 mg/kg, i.p.) or saline (1 ml/kg, i.p.) on alternating morning and afternoon sessions with a 6-h interval. In the test (Day 7), the mice moved freely in the chamber, and the time spent in each compartment was recorded. The CPP scores (sec) were identified as the time spent in the morphine-paired chamber minus the time spent in the saline-paired chamber.

### RNA Sequencing

Total RNA from the samples was isolated using TRIzol reagent. Sequencing libraries were generated using the NEBNextR UltraTM Directional RNA Library Prep Kit for IlluminaR (NEB, United States) following the manufacturer’s recommendations. Libraries were tested for quality and quantified using the Agilent Bioanalyzer 2,100 system (Agilent Technologies, CA, United States) and qPCR. Then, the library preparations were sequenced on an Illumina platform.

Clean data were acquired by eliminating adapter-containing reads, poly-N sequence-containing reads and low-quality reads from the raw data. These clean reads were then mapped to the reference genome sequence by HISAT2 (v2.0.4). Then, StringTie (v1.3.1) was used for assembling the transcriptome. The gffcompare program was used for annotating the assembled transcripts. CPC2 (CPC2-beta)/CNCI (v2)/Pfam (v1.3)/CPAT was used to separate the protein-coding genes from the non-coding genes. Furthermore, different classes of lncRNAs, such as lincRNAs, intronic lncRNAs, antisense lncRNAs, and sense lncRNAs, were identified using cuffcompare. lncLocator^1^ was used to calculate the subcellular localization of lncRNAs (21). Fragments per kilobase of exon per million fragments mapped (FPKM) of mRNAs and lncRNAs, and the read number of lncRNAs mapped, was calculated using StringTie. The DESeq R package (1.10.1) was used to analyze differential expression of lncRNA and mRNA between morphine group and saline group. DEmRNAs and differentially expressed lncRNAs (DElncRNAs) were identified by the criteria of |log2-fold change (FC) | ≥ 1.5 and adjusted *p* < 0.01.

### Small RNA Sequencing

A total amount of 3 μg of total RNA per sample was used for the small RNA library. First, the 3 ′SR and 5′SR adaptors were ligated. Then, reverse transcription is performed to synthesize the first chain. Finally, PCR products were purified (AMPure XP system). DNA High Sensitivity Chips was used to was assess the Library quality. The library preparations were sequenced on an Illumina HiSeq 2500/2000 platform.

Clean data were acquired by eliminating adapter-containing reads, poly-N sequence-containing reads and low-quality reads. Reads with a length < 15 nt and > 35 nt were removed. Bowtie tools software, The Clean Reads with Silva database,^2^ GtRNAdb database,^3^ Rfam database^4^ and Repbase database^5^ sequence alignment, filter ribosomal RNA, transfer RNA, small nuclear RNA, small nucleolar RNA and other ncRNA and repeats. The remaining readings were compared to known miRNAs in miRBase^6^ to discover known miRNAs and novel miRNAs predicted. Randfold (v2.1.7) was used to predict novel miRNA secondary structure. The expression levels of miRNAs were estimated for each sample. Only the miRNAs with the criteria of |log2FC (fold change) | ≥ 0.58 and *p* ≤ 0.05 were identified as differentially expressed miRNAs (DEmiRNAs).

### Construction of the Competing Endogenous RNA Network

CircRNAs, lncRNAs, and mRNAs, as competing endogenous RNAs (ceRNAs), can indirectly modulate gene expression by competitively binding target gene regulation of miRNAs. According to the ceRNA hypothesis, the lncRNA-miRNA-mRNA network was constructed and visually shown using Cystoscope software V3.5.0 (San Diego, CA, United States). Different colors indicate different RNA types.

### Gene Ontology and Kyoto Encyclopedia of Genes and Genomes Analysis

Gene Ontology (GO) enrichment analysis of the differentially expressed genes was implemented by the GOseq R package. KEGG analysis was implemented by KEGG database.^7^ KOBAS software was used to test the statistical enrichment of differentially expressed genes in KEGG pathways.

### Quantitative Reverse Transcription Polymerase Chain Reaction

Nucleus accumbens tissues were treated with the miRNAeasy Mini Kit (Qiagen, United States) to isolate total RNA, according to the manufacturer’s instructions. Data was analyzed using ABI Prism 7500 sequence detection system software. A total of 1.0 μg of RNA was used for reverse transcription (RT) with the PrimeScript RT reagent Kit (TaKaRa, China) or Mir-X miRNA First-Strand Synthesis Kit (TaKaRa, China). Real-time PCR was performed using TB Green Premix Ex Taq II (TaKaRa, China) or TB Green ^®^ Advantage ^®^ qPCR Premix (TaKaRa, China). For lncRNA and mRNA normalization, GAPDH was employed as an internal reference, whereas U6 was used for miRNA normalization. The expression levels of lncRNA, mRNA and miRNA are represented as fold changes using the 2^–ΔΔCt^. The primers are listed in Supplementary File 1.

### Data and Statistical Analyses

Data are analyzed using Prism 8.0 software, and are expressed as the means ± SEM. The statistical analyses were performed using Student’s *t*-test or two-way repeated-measures ANOVA for each experiment followed by the Bonferroni post-test. *p* < 0.05 was defined significant.

## Results

### Establishment of the Morphine Conditioned Place Preference Model

**FIGURE 1:**
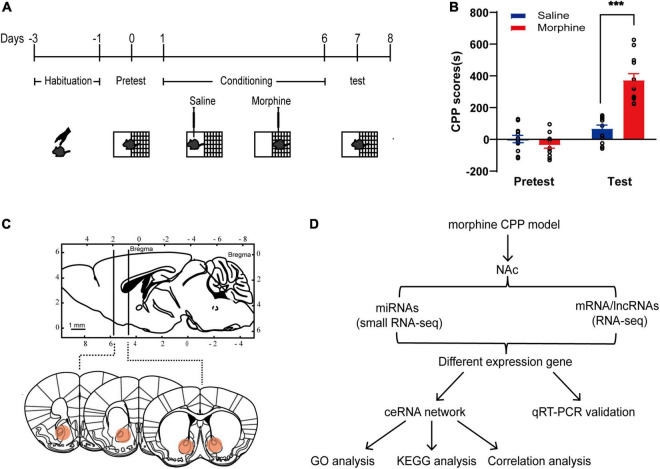
Establishment of the morphine CPP model. **(A)** Chematic experimental design for the training and test of morphine-CPP. **(B)** CPP scores of saline- and morphine-treated mice on Days 0 and 7. The data are presented as the mean ± SEM, *n* = 12, ***p < 0.001, compared with the saline group. **(C)** Up: The position of the razor blades used to cut the nucleus accumbens (NAc) are shown in a sagittal section of the mouse brain; down: Schematic illustrations of NAc location in coronal slices (red area). **(D)** Flowchart for the identification and analysis of ncRNAs and mRNAs in the ceRNA network.

The classical CPP paradigm was used to establish drug-associated memory (19). The mice were treated with 10 mg/kg morphine or saline for 6 day, and the CPP test was performed 24 h after the last session (Figure 1A). Statistical analysis (two-way repeated-measures ANOVA) demonstrated a significant effect from the interaction [*F*_(1, 22)_ = 34.95, *p*-value < 0.001] between the treatments (saline vs. morphine) and the tests (pretest vs. test). Significant differences were found between saline- and morphine-conditioned mice [*F*_(1, 22)_ = 66.28, *p*-value < 0.001] and within the pretest and the test [*F*_(1, 22)_ = 23.79, *p*-value < 0.001]. The Bonferroni *post-hoc* test revealed that morphine-treated mice spent significantly more time in the drug-paired context during the test than saline-treated mice (*t* = 7.647, *p*-value < 0.001; Figure 1B). For further correlative analysis, the NAc tissues were collected at half an hour after the test (Figure 1C).

### Differential Expression Analysis: Morphine Group vs. Saline Group

**FIGURE 2:**
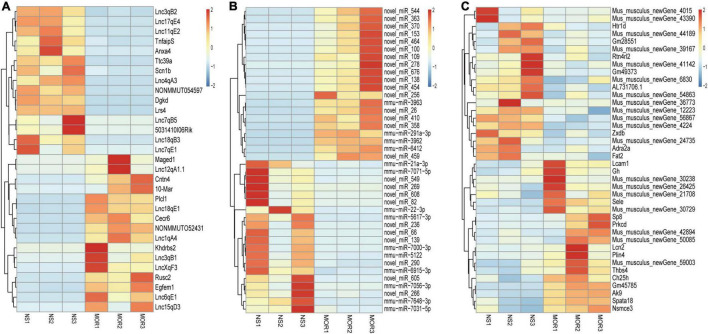
Heatmaps of differentially expressed lncRNA **(A)**, miRNA **(B)**, and mRNA **(C)** profiles. Unsupervised clustering analysis show the expression profiles of differentially expressed lncRNAs, miRNAs (top 40), and mRNAs (top 40) in the morphine group compared to saline group. MOR1, MOR2, and MOR3 represent the NAc tissue of three mice treated with morphine. NS1, NS2, and NS3 represent the NAc tissue of three mice treated with saline. Red indicates upregulation, and blue indicates downregulation.

As shown in the schematic diagram in Figure 1D, lncRNA-mRNA RNA-Seq and miRNA-Seq were used to capture the expression profiles of ncRNAs and mRNAs in the morphine group and saline group. According to the criteria of a |log2 (fold change)| ≥ 1.5 and a *p*-value < 0.01, 31 DElncRNAs (16 up- and 15 downregulated) and 271 DEmRNAs (168 up- and 103 downregulated) were identified in the morphine group compared with the saline group (Supplementary Figures 1A,C). A heatmap was constructed to visualize the cluster analysis results of DElncRNA and DEmRNA expression (Figures 2A,C). Detailed information on the DERNAs is listed in Supplementary Tables 1, 2.

Regarding the DEmiRNAs, the changes in 393 miRNAs met the criteria |log2FC (fold change) | ≥ 0.58 and *p*-value ≤ 0.05. Detailed information about the 245 upregulated and 148 downregulated miRNAs (Supplementary Figure 1B), which were significantly altered between the morphine group and the saline group, is listed in Supplementary Table 3. Figure 2B shows a heatmap of the DEmiRNA expression profile. An overview of differential RNA expression across the genome is given in Supplementary Figure 2.

### Quantitative Reverse Transcription Polymerase Chain Reaction Validation

**FIGURE 3:**
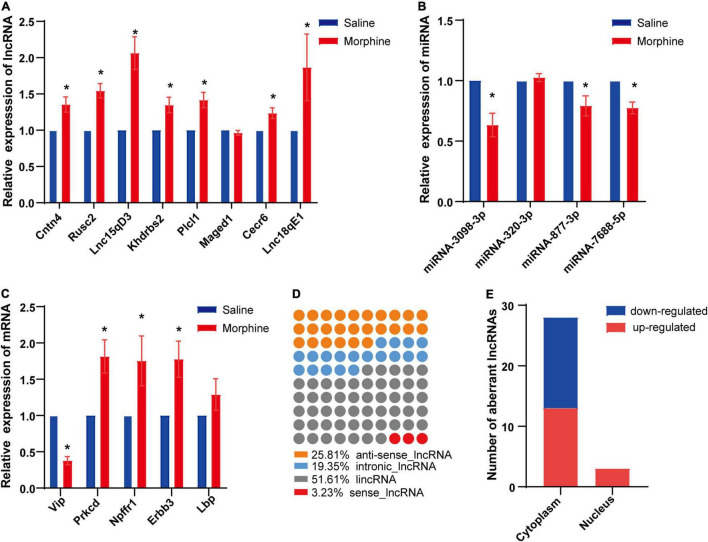
Differential expression of distinct RNAs and characteristics of LncRNAs. The relative expression levels of eight lncRNAs **(A)**, four miRNAs **(B)** and five mRNAs **(C)** are shown by comparing the morphine group with the saline group *via* qRT-PCR. The data are presented as the mean ± SEM, *n* = 4, *p < 0.05. Subclasses **(D)** and subcellular localization **(E)** of significantly dysregulated lncRNAs were analyzed.

To confirm the reliability of the RNA-seq data, 10 DElncRNAs, 4 DEmiRNAs and 6 DEmRNAs were randomly selected and verified by qRT-PCR. The results showed that 7 of 8 DElncRNAs (Cntn4, Rusc2, Lnc15qD3, Khdrbs2, Plcl1, Cecr6, and Lnc18qE1), 3 of 4 DEmiRNAs (miRNA-3098-3p, miRNA-877-3p, and miRNA-7688-5p) and 4 of 5 DEmRNAs (Vip, Prkcd, Npffr1, and Erbb3) exhibited significant differences between the saline and morphine groups (Figures 3A–C).

### Class Distributions of the Differentially Expressed lncRNAs

Long non-coding RNAs have been divided into several classes based on their genomic position in relation to coding genes, which aids in predicting some of their potential mode of action in modulating other cellular RNA molecules (22). Based on this classification, DElncRNAs in the morphine group compared to the saline group were discovered to be 52% (16) intergenic, 26% (8) natural antisense, 19% (6) intronic antisense regions and 3% (1) sense (Figure 3D) in the genome. Like proteins, the function of lncRNAs is dependent on their subcellular localization (23). Therefore, the subcellular localization of DElncRNAs was predicted using lncLocator. Our findings indicated that among the DElncRNAs, cytoplasm accounted for the majority, and very few DElncRNAs were located in the nucleus (Figure 3E).

### Construction of a Long Non-coding RNA-MicroRNA-mRNA Network

**FIGURE 4:**
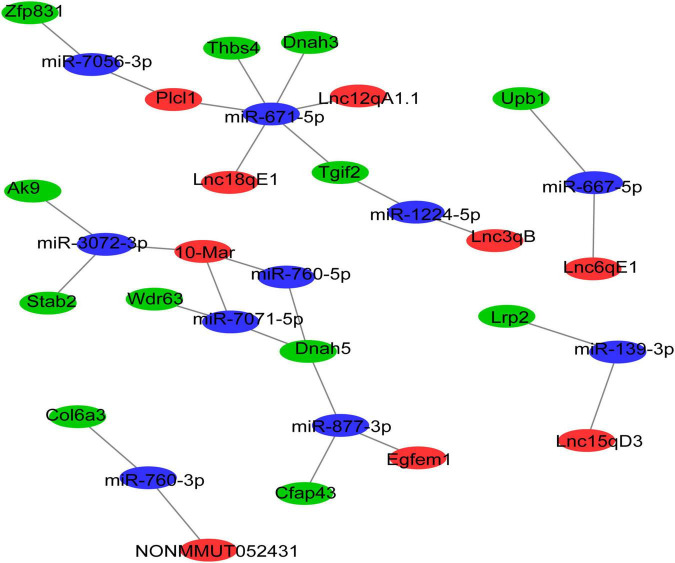
The lncRNA-associated ceRNA networks in morphine/saline treated mice. The ceRNA network was constructed based on identified lncRNA–miRNA and miRNA–mRNA interactions. In the network, red indicates upregulated lncRNAs, blue indicates downregulated miRNAs, and green indicates upregulated mRNAs.

Cytoplasmic lncRNAs can regulate gene expression by serving as decoys for miRNAs (24). Thus, the differentially expressed ceRNA network was established, in which DElncRNAs were used as the center to extract DEmRNAs and DEmiRNAs. Finally, 9 DElncRNAs, 9 DEmiRNAs, and 10 DEmRNAs were involved in the differentially expressed ceRNA network (Figure 4). The differentially expressed ceRNA networks only included one situation: lncRNA (upregulated in morphine group)–miRNA (downregulated in morphine group)–mRNA (upregulated in morphine group).

**FIGURE 5:**
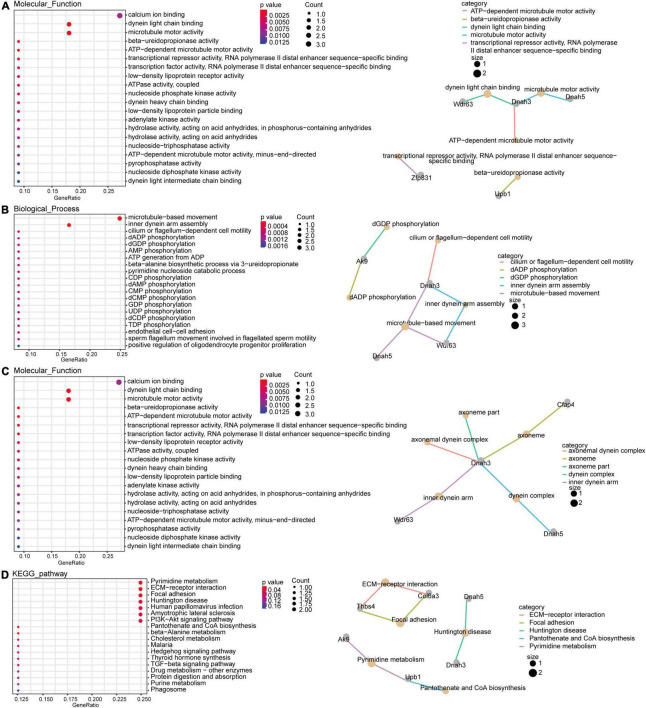
Gene Ontology (GO) and KEGG pathway function enrichment analysis for DEmRNAs. The significant molecular functions **(A)**, biological processes **(B)** and cellular components **(C)** of DEmRNAs in the ceRNA network were presented using GO analyses. Pathway analyses results of DEmRNAs in the ceRNA network with fold changes >1.5 **(D)**.

To explore the molecular functions of the ceRNA network, DEmRNAs of the ceRNA network were analyzed by GO and KEGG analysis. The GO results indicated that the most significantly enriched molecular functions of the DEmRNAs in the ceRNA network were dynein light chain binding, beta-ureidopropionase activity, and biological processes focused on inner dynein arm assembly and microtubule-based movement (Figures 5A,B). The cell components mainly concentrated on the inner dynein arm, dynein complex and axoneme (Figure 5C). The pathway analyses revealed that the most significantly enriched pathways of DEmRNAs included pyrimidine metabolism, ECM-receptor interaction and focal adhesion (Figure 5D).

### Regression Analyses of Long Non-coding RNA Related Competing Endogenous RNA Network

**FIGURE 6:**
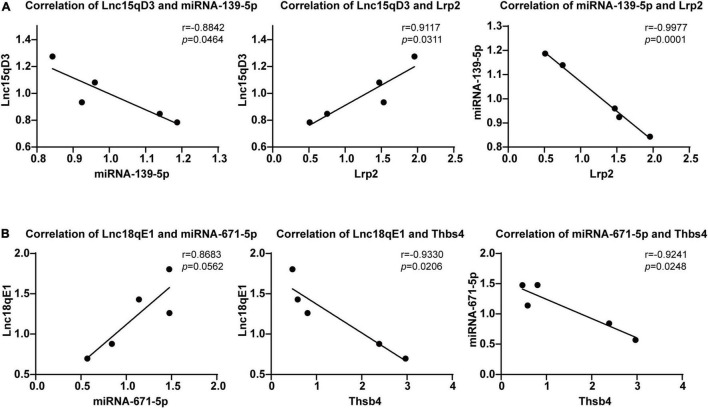
Regression analyses of the ceRNA network. The correlations of Lnc18qE1-miR-671-5p-Thbs4 **(A)** and Lnc15qD3-miR-139-3p-Lrp2 **(B)** were determined by qRT-PCR.

To select the ceRNA network we are concerned, we set two screening criteria. One is to select lncRNA related ceRNA, which differential expression between the morphine group and saline group was confirmed by qRT-PCR; moreover, the target genes of lncRNA which are involved in synaptic plasticity pathway were considered as another screening criterion. According to these conditions, we focused on two ceRNA networks, Lnc18qE1-miR-671-5p-Thbs4 and Lnc15qD3-miR-139-3p-Lrp2. To further verify the prediction, regression analyses were conducted between the expression levels of the ncRNAs and mRNAs in the concerned ceRNA network. The correlations of Lnc15qD3-miR-139-3p-Lrp2 and Lnc18qE1-miR-671-5p-Thbs4 were consistent with the bioinformatics analysis (Figures 6A,B), and the reverse was true for Lnc18qE1 and miR-671-5p (Figure 6B). These results suggested that Lnc15qD3-miR-139-3p-Lrp2 might exhibit ceRNA activity in morphine addiction.

## Discussion

Opioids addiction is a complex neuropsychiatric disorder that leads to extremely serious public health problems. Numerous progresses have been made in identifying opioids addiction-related molecular and cellular processes (25), but the molecular mechanisms underlying opioids addiction remain largely unclear. Recent data suggest that lncRNAs play a key role in neuropsychiatric disorders (12). However, the roles of lncRNAs in morphine addiction need to be clarified. Here, we obtained lncRNA, miRNA and mRNA transcriptome profiles that were aberrantly expressed in the morphine group by RNA-Seq. Then, we constructed a ceRNA network of reciprocal interactions for 9 DElncRNAs, 9 DEmiRNAs, and 10 DEmRNAs in morphine addicted mice using bioinformatics tools. Furthermore, DEmRNAs in the ceRNA network were highly enriched in synapse-related pathways, such as the PI3K-Akt signaling pathway, according to GO and KEGG analyses. Finally, the relation of Lnc15qD3-miR-139-3p-Lrp2 was validated by qRT-PCR and correlation analysis, which matched the predicted ceRNA hypothesis.

We firstly identified DElncRNAs, DEmiRNAs and DEmRNAs in morphine addiction. Although these lncRNAs have not been reported to be involved in drug addiction, dysregulated expression of lncRNAs has been reported to play a critical role in the progression of addiction (26, 27). Gao et al. showed that upregulation of MEG3 was involved in morphine-induced autophagy in hippocampal neuronal HT22 cells (28). In addition, analysis of significantly dysregulated miRNAs revealed many downregulated miRNAs, including miR-124 and miR-218, and upregulated miRNAs, including miR-451a and miR-15b-3p, were involved in drug addiction. Guo et al. suggested that miR-124 played a role in cocaine-mediated microglial activation and neuronal adaptations (29). Chandrasekar et al. determined that the regulation of miR-124 in the accumbens influences the expression, extinction, and reinstatement of cocaine-induced addiction memory (30). Moreover, these profiles revealed by RNA-seq were further confirmed by qRT-PCR validation, thus indicating the reliability of the RNA-seq data. Although the expression of a few RNAs is inconsistent with the RNA-seq results, the heterogeneity of the diverse samples and the sequencing of a small number of samples in our investigation might explain this conclusion. Collectively, these results suggested that these transcripts might be associated with the pathogenesis of morphine addiction.

Long non-coding RNAs have biochemical diversity and function in diverse lncRNA gene locations (31) and diverse cellular contexts (23). Specifically, lncRNAs can modulate the expression of nearby genes (acting in *cis* in the nucleus) (32) or genes elsewhere in cells (acting in trans in the nucleus or cytoplasm) (23). Cytoplasmic lncRNAs can interfere with protein post-translational modifications, resulting in aberrant signal transduction (33) and influencing gene regulation by acting as decoys for miRNAs (24) and proteins (34). Among these processes, the ceRNA hypothesis, as ceRNAs to regulate the activities and biological functions of miRNAs, has been reported to be the main function of several cytoplasmic lncRNAs. Weng et al. showed that lncRNA-1604, which is highly expressed in the cytoplasm, orchestrates neural differentiation through the miR-200c/ZEB axis (35). In our study, cytoplasmic intergenic lncRNAs (lincRNAs) accounted for the majority among these DElncRNAs. Thus, the potential function of these lncRNAs might be as ceRNAs. Next, we constructed a ceRNA network of reciprocal interactions for DEmiRNAs, DElncRNAs, and DEmRNAs in morphine addiction. An increasing number of studies have indicated that the ceRNA hypothesis plays a role in the pathogenesis of many diseases, although our regulatory network has never been evaluated. Chen et al. indicated that MALAT1 regulated the chemoresistance of lung adenocarcinoma (LUAD) cells by sponging miR-200b to regulate E2F3 and ZEB1 (36). Furthermore, several lncRNA-related ceRNAs have been reported in nervous system disorders. Ge et al. determined that interference with BACE1-AS-regulated ceRNA networks could alleviate neuronal damage (17). Xu et al. showed that lncGAS5 promotes PD progression *via* regulating the miR-223-3p/NLRP3 axis (18). To further validate the credibility of the ceRNA hypothesis, the correlation of ceRNA pairs was analyzed. Our findings revealed that the correlation of the lnc15qD3-related ceRNA network in morphine addiction tissue samples was highly consistent with bioinformatics prediction. Of these mRNAs in our concerned ceRNA network, THBS4 directly act on neurons as synaptogenic factors and may represent rejuvenation factors that enhance synaptic connectivity by increasing dendritic arborization, synapse formation, and synaptic transmission (37). And LRP2 (low-density lipoprotein receptor 2/megalin) is also expressed in the CNS, mainly in neurons, being involved in neurite outgrowth and neuroprotective mechanisms (38). In addition, multiple studies suggest miR-671-5p may be involved in neuropsychiatric diseases, such as PD pathogenesis (39). Overall, these lncRNA-related ceRNA triplets might contribute to the formation of morphine addiction.

Finally, GO and KEGG pathway analyses were performed on dysregulated mRNAs in the ceRNA to identify the important pathways in morphine addiction, most of which are classic pathways that play critical roles in synaptic plasticity, such as the PI3K-Akt signaling pathway. Brain-derived neurotrophic factor (BDNF), one of PI3K-Akt signaling pathway molecules, has been reported to play a key role in morphine-dependent behaviors (40). In particular, several studies suggest a promoting effect of BDNF in the NAc on morphine-induced CPP and morphine-primed reinstatement (41, 42). Furthermore, some of the other novel biological processes and pathways might be involved in morphine addiction formation and development, such as inner dynein arm assembly, ECM-receptor interaction and focal adhesion, which supplements the findings of previous studies. Of course, further evidence should be acquired to clarify the identified function of these pathways in morphine addiction. Altogether, these findings reveal that the molecular mechanism of the ceRNA network could be involved in the progression of morphine addiction and may become a therapeutic target of morphine addiction.

There are also some limitations in this study. The NAc is neuroanatomically divided into the NAc core and shell, which are heterogeneous structures with different afferent and efferent connections (43) and may underlie the dissociable role in the conditioned reinforcing effects of drug-associated cues (44). Liang et al. reported that glutamate receptor interacting protein 1 (GRIP1) regulated the reconsolidation of drug cue memories in the NAc core but not the shell (45). However, RNA-seq was not conducted for the NAc core and shell separately due to the sample size being too small. Therefore, the expression and function of these transcripts in different subregions of the NAc need to be further verified. Moreover, *in vivo* and *in vitro* experiments are needed in further for validation of multiple interactions of miRNAs, lncRNAs and mRNAs.

Taken together, we first characterized differentially expressed ncRNAs and mRNAs in the NAc after morphine-induced CPP and constructed a ceRNA network based on validated reciprocal interactions between DEncRNAs and DEmRNAs in morphine addiction. The current findings show new light on the lncRNA-related ceRNA network in morphine addiction and identify potential diagnostic and prognostic biomarkers.

## Data Availability Statement

The datasets presented in this study can be found in online repositories. The names of the repository/repositories and accession number(s) can be found below: https://www.ncbi.nlm.nih.gov/, GSE199917 and https://www.ncbi.nlm.nih.gov/, GSE200282.

## Ethics Statement

The animal study was reviewed and approved by the Animal Care and Use Committee of Hebei Medical University.

## Author Contributions

BX, DW, CM, and BC were held responsible for the study design and supervision. XL, YL, LZ, JW, HY, and FY had involved in the acquisition and analysis of the data. XL wrote the first draft of the manuscript. BX and DW revised the manuscript for essential intellectual content. All authors contributed to the article and approved the final manuscript.

## Conflict of Interest

The authors declare that the research was conducted in the absence of any commercial or financial relationships that could be construed as a potential conflict of interest.

## Publisher’s Note

All claims expressed in this article are solely those of the authors and do not necessarily represent those of their affiliated organizations, or those of the publisher, the editors and the reviewers. Any product that may be evaluated in this article, or claim that may be made by its manufacturer, is not guaranteed or endorsed by the publisher.

## Footnotes

1
http://www.csbio.sjtu.edu.cn/bioinf/lncLocator/

2
http://www.arb-silva.de/

3
http://lowelab.ucsc.edu/GtRNAdb

4
http://rfam.xfam.org/

5
http://www.girinst.org/repbase/

6
http://www.mirbase.org/

7
http://www.genome.jp/kegg/
