# Supplementary material for: Transcriptomic Analysis of Long Non-coding RNA-MicroRNA-mRNA Interactions in the Nucleus Accumbens Related to Morphine Addiction in Mice
Source: Front Psychiatry. 2022 Jun 2;13:915398. doi: 10.3389/fpsyt.2022.915398 (PMC9201067; doi:10.3389/fpsyt.2022.915398)
Supplement: Supplementary file 1 [file Data_Sheet_1.docx]

Supplementary Material

## Supplementary Figures


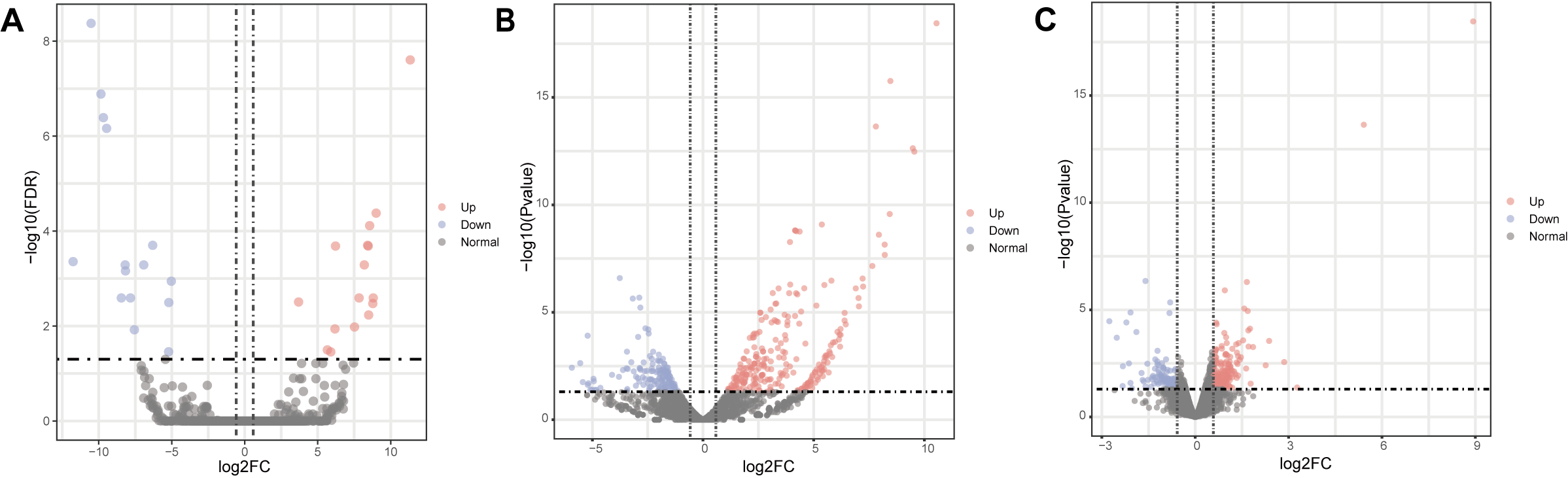


**Supplementary Figure 1**

**
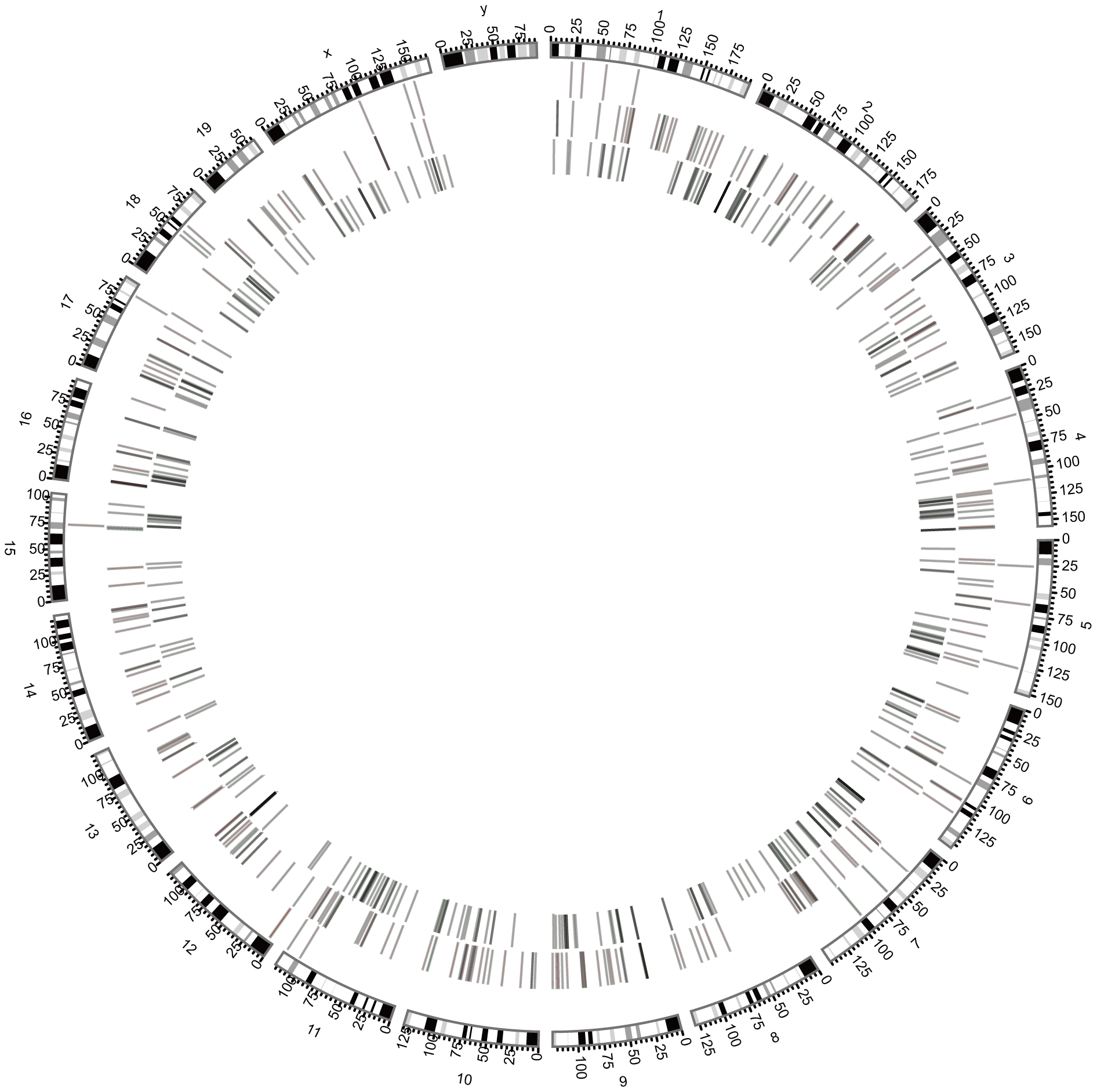
**

**Supplementary Fig 2**

**Supplementary Figure 1.** Volcano plot of differentially expressed lncRNA **(A)**, miRNA **(B)** and mRNA **(C)** profiles.

Volcano plot of DElncRNAs **(A)**, DEmiRNAs **(B)**, and DEmRNAs **(C)** expression profiles between morphine group and saline group. Red indicates upregulation, and blue indicates downregulation.

**Supplementary Fig 2.** Circos plot incorporating differential lncRNA, mRNA and miRNA expression.

Chromosome numbers and bands are identified in the outer-most ring. Other tracks from outer to inner represent: Chromosomal positions of differentially expressed lncRNA, differentially expressed mRNA and differentially expressed miRNA between control and morphine group, respectly. Red in the circle chart for each difference group indicates up, green for down, and high for significance (-log10 (FDR or P Value)).
